# expam—high-resolution analysis of metagenomes using distance trees

**DOI:** 10.1093/bioinformatics/btac591

**Published:** 2022-08-27

**Authors:** Sean M Solari, Remy B Young, Vanessa R Marcelino, Samuel C Forster

**Affiliations:** Centre for Innate Immunity and Infectious Diseases, Hudson Institute of Medical Research, Clayton, VIC 3168, Australia; Department of Molecular and Translational Science, Monash University, Clayton, VIC 3168, Australia; Centre for Innate Immunity and Infectious Diseases, Hudson Institute of Medical Research, Clayton, VIC 3168, Australia; Department of Molecular and Translational Science, Monash University, Clayton, VIC 3168, Australia; Centre for Innate Immunity and Infectious Diseases, Hudson Institute of Medical Research, Clayton, VIC 3168, Australia; Department of Molecular and Translational Science, Monash University, Clayton, VIC 3168, Australia; Centre for Innate Immunity and Infectious Diseases, Hudson Institute of Medical Research, Clayton, VIC 3168, Australia; Department of Molecular and Translational Science, Monash University, Clayton, VIC 3168, Australia

## Abstract

**Summary:**

Shotgun metagenomic sequencing provides the capacity to understand microbial community structure and function at unprecedented resolution; however, the current analytical methods are constrained by a focus on taxonomic classifications that may obfuscate functional relationships. Here, we present *expam*, a tree-based, taxonomy agnostic tool for the identification of biologically relevant clades from shotgun metagenomic sequencing.

**Availability and implementation:**

*expam* is an open-source Python application released under the GNU General Public Licence v3.0. *expam* installation instructions, source code and tutorials can be found at https://github.com/seansolari/expam.

**Supplementary information:**

Supplementary data are available at *Bioinformatics* online.

## 1 Introduction

Microbial communities perform essential functions in a variety of ecosystems (Danovaro *et*  *al.*, 2008) including the human body (Lloyd-Price *et al.*, 2017), where compositional changes have been correlated with diseases from inflammatory bowel disease (Ni *et al.*, 2017) to cancers (Frankel *et al.*, 2017) and autoimmune diseases (Brown *et al.*, 2011). Shotgun metagenomic sequencing now represents the gold-standard for rapid assessment of the functional capacity and composition of these microbial communities. Applying the reference-based metagenomic analysis to these datasets (Beghini *et al.*, 2021; Brady and Salzberg, 2009; LaPierre *et al.*, 2020; Milanese *et al.*, 2019; Wood *et al.*, 2019), shotgun reads are compared against sequence collections to ascertain the taxonomic distribution of species within the community (Forster *et al.*, 2019; Lloyd-Price *et al.*, 2017). While taxonomy provides an important standard for describing and comparing microbes, prokaryotic taxonomic groups do not necessarily capture precise genomic relationships (Fraser *et al.*, 2009). Specifying the resolved hierarchical structure between reference genomes enables clade-specific functional associations, thereby facilitating an ability to understand phenotypic relationships at a resolution lost using taxonomy. Here, we implement this concept in a software tool called *expam. expam* provides precise phylogenetic profiling of metagenomic data using highly resolved trees, simultaneously analysing shotgun data for signs of novel biological sequence.

## 2 Materials and methods

### 2.1 expam database

Construction of the *expam* database requires two sources of data: a collection of reference sequences, and a Newick tree specifying their relationship. Optimal classification performance requires accurate, high-resolution trees; while tree specification is left at the user’s discretion, this criterion makes distance-based and phylogenetic trees primary candidates.

**Fig. 1.**
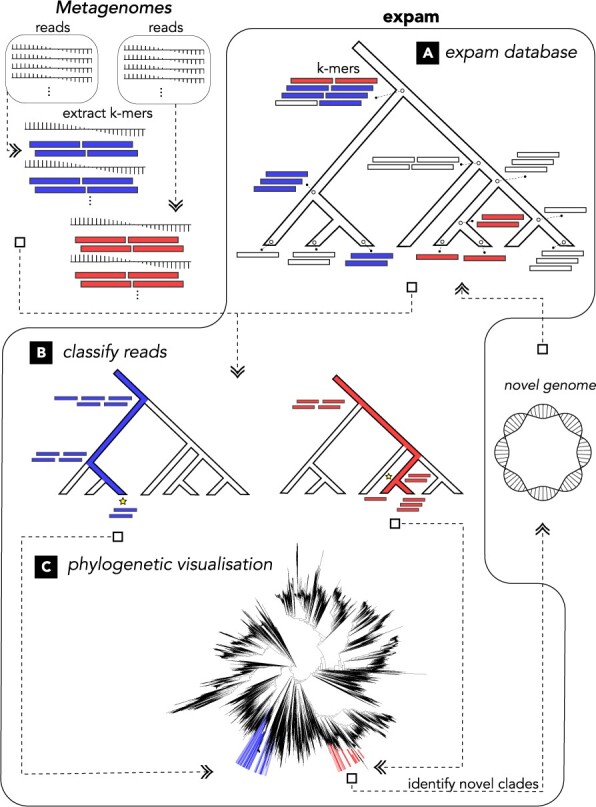
Overview of the *expam* pipeline using two synthetic metagenomes. (**A**) *k*-mers are extracted from each metagenomic read and mapped against an *expam* database. (**B**) The *k*-mer distribution of this read is analysed and classified within the reference tree (gold stars). (**C**) Reads classifications are accumulated, and the phylogenetic distribution of various samples can be plotted and compared

Like many *k*-mer-based metagenome profilers, the database consists of a key-value store, with each key being a *k*-mer from some reference sequence. However, each database value now refers to that node within the tree which is the lowest common ancestor (LCA) of all reference sequences containing the corresponding key, rather than the shared taxonomic ancestor (Fig. 1A). To construct this database, *expam* uses Python multiprocessing to concurrently extract and sort *k*-mers (Knuth, 1998; Marcais and Kingsford, 2011) from all reference sequences, before then mapping these *k*-mers to their LCA. The resulting *k*-mer and LCA *NumPy* arrays (Harris *et al.*, 2020) are compressed on disk using the *PyTables* library, and loaded into shared memory during sample processing for parallel read classification.

### 2.2 Classification algorithm

Within the highly resolved tree, each read has some *k-mer distribution*, or the set of nodes that *k*-mers from this read are mapped to. The *k*-mer distribution of any sequence present in some reference *S* must lie within the root-to-leaf path terminating at *S*. Metagenomic reads can therefore present either as *single-lineage* (SL) *reads*, or *split-lineage reads* (hereafter *splits)*, whose *k*-mers are distributed along one or multiple lineages, respectively. In both cases, reads are assigned to the lowest common node of all lineages (Fig. 1B). However, high *split* counts in a particular region of the tree suggest the presence of microbial isolates lacking reference genomes in the database. The inclusion of specific reference sequences belonging to these under-represented clades can therefore enable targeted classification improvement. A heuristic α parameter filters low abundance lineages in the k-mer distribution (Supplementary Equation S1), such as those arising from sequencing error. The default α parameter value is suitable for general use cases. Finally, identified clades from each sample are available as raw counts in standard Kraken output format and visualized by *expam* in the reference phylogenetic tree (Fig. 1C).

### 2.3 Converting classifications to NCBI taxonomy

Despite the disadvantages of taxonomy for read classification, it remains a valuable tool for the communication of findings. To obtain a taxonomic summary of tree-based classifications, *expam* maps each point in the reference tree to the LCA of all taxonomic lineages among reference sequences below this point. These results are output in the same standardized Kraken output format.

## 3 Results

We compared *expam’*s performance to a collection of widely used metagenomic profilers (Beghini *et al.*, 2021; Gruber-Vodicka *et al.*, 2020; Marcelino *et al.*, 2020; Müller *et al.*, 2017; Wood *et al.*, 2019) (Supplementary Table S1) on 140 publicly available simulated metagenomic communities (Parks *et al.*, 2021), stratified by four distinct classes: either low or high species diversity, and single or multiple strains (Supplementary Table S2). To standardize classifier performance, the RefSeq collection (release 203) of genome sequences was used as a reference for all software with the capacity to build a custom database, default databases being used for *phyloFlash* and *MetaPhlAn3*. Read-level analysis of classifier performance was used to determine the assignment accuracy of each read, and taxonomic summaries assessed the total set of taxa estimated to be in the sample (Supplementary Methods).

Our results demonstrate that *expam* achieves stringent taxonomic and read-level species precisions of 84.0% and 63.9%, respectively, when averaged across the 140 samples (Supplementary Figs S1, S2, and Table S3) exhibiting a robustness to spurious read classifications (Anyasi *et al.*, 2020) that contrasted the results of *Kraken2* (read-level 74.1%; taxonomic 4.1%) and *MetaCache* (read-level 86.9%; taxonomic 11.1%). Of all tools using the standardized database, *expam* achieves the highest average species-level taxonomic F1 score of 0.575, with the next highest score 0.211 achieved by *CCMetagen* (Supplementary Figs S3 and S4). Notably, *expam* achieved an average taxonomic recall of 55.8%, a 23% decrease from the top recall score (Kraken2, 72.2%) (Supplementary Figs S5 and S6); however, *expam’*s taxonomic recall generally depends on the degree to which the reference tree and NCBI taxonomy align.

To gauge sensitivity of runtime statistics to *k*-mer length and number of reference genomes, a collection of six *expam* databases were built varying number of reference sequences and *k*-mer length (Supplementary Tables S4–S7) before being tested against simulated metagenomes. While precision and recall increased with references, build and classification memory also increased with the amount of reference sequence (Supplementary Fig. S7). Classification time and memory usage were relatively stable for larger *k*, being determined predominantly by number of references (Supplementary Tables S4–S7); however, a large *k*-mer length relative to the number of reference genomes hinders recall (Supplementary Fig. S8). A pre-built *expam* database is made publicly available to overcome the comparatively large computational resources required for database indexing (see *Data Availability*).

The distance tree-based method employed by *expam* achieves a resolution that matches existing approaches when translated into the taxonomic space while increasing the discriminative power of metagenomic analysis to taxonomy agnostic isolate and clade analysis. This approach provides the ability for targeted analysis including high-resolution assessment and correction of database coverage and clade-specific functional analysis.

## Supplementary Material

btac591_Supplementary_Data
