# Supplementary material for: expam—high-resolution analysis of metagenomes using distance trees
Source: Bioinformatics. 2022 Aug 27;38(20):4814–6. doi: 10.1093/bioinformatics/btac591 (PMC9563691; doi:10.1093/bioinformatics/btac591)
Supplement: btac591_Supplementary_Data [file btac591_supplementary_data.pdf]

Supplementary Materials: expam - high-resolution analysis of metagenomes using distance trees

Sean M. Solari*[1,2], Remy B. Young*[1,2], Vanessa R. Marcelino[1,2], Samuel C. Forster[1,2]

1. Centre of Innate Immunity and Infectious Diseases, Hudson Institute of Medical Research, Clayton 3168, Australia.

2. Department of Molecular and Translational Science, Monash University, Clayton 3168, Australia.

*These authors contributed equally.

Supplementary Information

The expam v1.0.4 RefSeq Database used for benchmarking, expam v1.0.4 source code used for benchmarking, Python scripts used for profiling classifier performance and excel document containing raw values for the performance summary statistics can be found at https://dx.doi.org/10.26180/c.5974267.

Supplementary Methods

1. Classification Algorithm and α parameter

Let $\mathcal{L} = \{\ell_i\}_i$ be the collection of lineages obtained by mapping the N (non-unique) k-mers of some read R, such that each lineage can be represented as an ordered tuple of nodes processing from the root down a single lineage $\ell_i = (\text{root}, n_1, n_2, \dots, n_{\ell_i})$. Let $|\ell_i|$ be the number of (non-unique) mapped k-mers contained in lineage ℓ_i, let $\alpha \in [0,1]$ be some real-valued parameter and LCN the *lowest common node* operator. With these definitions, the classification algorithm is defined by Equation S1.

Equation S1: Classification algorithm

$$\mathcal{L} \mapsto \text{LCN}\left\{\ell_i \middle| \ell_i \in \mathcal{L}, \frac{|\ell_i|}{N} \geq \alpha\right\}.$$

2. Reference Sequences

All RefSeq (release 203) complete assemblies of bacterial, archaeal and viral genomes were downloaded from NCBI ftp (https://ftp.ncbi.nlm.nih.gov/).

Reference sequences were edited to include their respective NCBI Taxonomy ID in the sequence header for input into Kraken2, Metacache and CCMetagen database building. The default reference databases were used for Phyloflash and MetaPhlAn3 as these tools use marker genes.

3. Database Indexing

Databases were built for Kraken2 v2.1.2, CCMetagen v1.4.0, MetaCache v2.0.0, phyloFlash v3.4, MetaPhlAn v3.0.12 and expam v1.0.4 using multiple threads/process if multithreading was available.

To build the reference database for each tool, the following commands were used:

expam:

1. `expam create -db refseq`
2. `expam set -k 31 -n 12 -db refseq -p refseq/phylogeny/tree.nwk`
3. `expam add -db refseq -d refseq_archaea/ -d refseq_viruses/ -d refseq_bacteria/`
4. `expam build -db refseq`

Kraken2:

```
kraken2-build --build --threads 12 --db Kraken2_db
```

MetaPhlAn3:

```
metaphlan --install --index mpa_v30_CHOCOPhlAn_201901 --bowtie2db
metaphlan_db
```

Phyloflash:

```
phyloFlash_makedb.pl --log PhyloFlashDB_log --CPUs 12 —remote
```

Metacache:

```
metacache build Metacache_db reference-sequences-folder -taxonomy
ncbi_taxonomy
```

CCMetagen:

```
kma index -i reference-sequences.fna -o kmaindex_db -NI -Sparse
TG -t 12
```

4. Test Data

140 *in silico* metagenomic communities generated by (Parks, 2021) (https://doi.org/10.5281/zenodo.4470159) were downloaded and used to assess the performance of metagenomic tools, including expam.

5. **Metagenomic classification**

Analyses with Kraken2, Metacache, MetaPhlAn3, CCMetagen, phyloflash and expam were performed with 32 threads/processes, where multi-threading was available. The following commands were used to perform metagenomic classification:

Kraken2

```
/usr/bin/time -v kraken2 --db Kraken2_db --paired sample_R1.fq.gz
sample_R2.fq.gz --threads 32 --report sample_report
```

Metacache

```
/usr/bin/time -v metacache query Metacache_db sample_R1.fq.gz
sample_R2.fq.gz -pairfiles -threads 32 -lowest species -lineage -
taxids -sample_result -abundances sample_abundances
```

MetaPhlAn3

```
/usr/bin/time -v metaphlan sample_R1.fq.gzsample_r9_R2.fq.gz --
input_type fastq --nproc 32 --bowtie2db bowtie_metaphlan_db --
bowtie2out sample_bt2_metaphlan.txt > sample_METAPHLAN_OUT.txt
```

PhyloFlash

```
/usr/bin/time -v phyloFlash.pl -lib sample-prefix -read1
sample_R1.fq.gz -read2 sample_R2.fq.gz -readlength 150 -dbhome
138.1 -CPUs 32 -taxlevel 7
```

CCMetagen

1. `/usr/bin/time -v kma -ipe sample_R1.fq.gz  sample_R2.fq.gz -o sample_result -t_db kma_db_1 -t 32 -1t1 -mem_mode -and -apm f -ef`
2. `/usr/bin/time -v CCMetagen.py -i sample_result.res -map sample_result.mapstat -o ccmetagen_sample_output`

expam

Using the default cutoff of 100 counts-per-million input reads:

```
/usr/bin/time -v expam_limit -x 0.8 -t 1.0 -o expam_classify.log
expam classify -db refseq --out samples140 -d
metagenomes/samples140 --taxonomy --alpha 0.1
```

6. Benchmarking

Metagenomic classifiers were assessed based on user time, elapsed wall time and maximum memory usage, as well as precision, recall and F1 score at the:

a. Taxonomic level

Taxonomic precision, recall and F1 score were calculated as follows.

$$\text{Precision} = \frac{True\ positive\ taxa}{True\ positive\ taxa + False\ positive\ taxa}$$

$$\text{Recall} = \frac{True\ positive\ taxa}{True\ positive\ taxa + False\ negative\ taxa}$$

$$\text{F1} = 2 \times \frac{Precision \times Recall}{Precision + Recall}$$

b. Read level

$$\text{Precision} = \frac{True\ positive\ reads}{True\ positive\ reads + False\ positive\ reads}$$

$$\text{Recall} = \frac{True\ positive\ reads}{True\ positive\ reads + False\ negative\ reads}$$

$$\text{F1} = 2 \times \frac{Precision \times Recall}{Precision + Recall}$$

Read level metrics were not calculated for MetaPhlAn3 and PhyloFlash; these tools use marker genes and only classify a subset of reads, such that recall cannot be accurately calculated.

False positive classifications are those absent in the truth data set, and false negatives are those that are present, but not identified in the classification. Additionally, F1 score is the weighted mean of precision and recall.

Analysis was performed using NCBI taxonomy IDs to compare tool assignment with truth data, at the following ranks: phylum, class, order, family, genus and species. All taxonomy IDs, from classifications and truth data, were updated using the ETE3 toolkit (Huerta-Cepas, 2016) to ensure all comparisons were made using the same taxonomy. Any incompatible taxids (those absent in the ETE3 toolkit local taxonomy database) were queried against the NCBI taxonomy database and mapped to the closest compatible parent taxid (scripts available at https://dx.doi.org/10.26180/c.5974267).

Custom python scripts were written to execute the above calculations on the 140 metagenomic classification outputs for each tool, available at https://dx.doi.org/10.26180/c.5974267.

For downstream analyses, the 140 *in silico* mock communities were grouped based on their complexity (number of species) and strain diversity (multiple strains of the same species).

7. Constructing a RefSeq distance tree

Using the same sketch-sizes and k-mer lengths to construct a distance tree containing the Archaeal, Viral and Bacterial RefSeq genomes resulted in inaccurate distance estimations. Distances trees were therefore constructed for these three domains separately, and subsequently joined in the following Newick topology: ((archaea, bacteria), viruses). Reference sequences in these domains was first sketched using the following commands (Tisza, 2021):

- `mash sketch -k 21 -s 50000 -p 10 -o bacteria.k21.s50000 bacteria/*.fna`

```
- mash sketch -k 21 -s 50000 -p 10 -o archaea.k21.s50000
  archaea/*.fna
- mash sketch -k 16 -s 5000 -p 10 -o virus.k21.s5000 virus/*.fna
```

Within each of these groups, pairwise distances were estimated using the following command:

```
- mash dist -p 10 -t *.msh *.msh > distances.tab
```

Neighbour-Joining was executed on this distance matrix, through the RapidNJ software, to obtain the final distance tree:

```
- rapidnj distances.tab -i pd -o t -c 10 > tree.nwk
```

8. Parameter Sensitivity Tests

A series of parameter sensitivity tests were undertaken to understand the impact of *k*-mer size and number of reference genomes on both database build time, classification runtime behaviour and classification performance. To test the impact of the number of reference genomes on *expam* database build behaviour, databases were built from two random subsets of 500 (hereafter *Ref500*) and 5,000 (hereafter *Ref5000*) reference genomes from the RefSeq (release 203) collection (hereafter *Ref29611*), keeping the same proportions of Archaeal, Bacterial and Viral references in each set. To test the impact of *k*-mer size on database build behaviour, Ref500 was built with three different *k* values: 11, 21 and 31. A reference tree for each subset of sequences was built using the method outlined in the previous section.

To test the classification runtime behaviour and performance of each of these databases, a subset of the MCP simulated metagenomes were classified, randomly taking two samples from each class outlined in Table S1: namely, ani100_cHIGH_stFalse_r3, ani100_cHIGH_stFalse_r7, ani100_cHIGH_stTrue_r1, ani95_cLOW_stFalse_r4, ani95_cLOW_stTrue_r1, ani97_cHIGH_stTrue_r8, ani99_cLOW_stFalse_r2 and ani99_cLOW_stTrue_r2. For each database and resulting sample classifications,

precision and recall were calculated according to methods and scripts outlined in the previous section.

Command used for database build:

- `/usr/bin/time -v expam_limit -t 10 -x 1.0 -o build.log expam build -db [DATABASE NAME]`

Command used for classification:

- `/usr/bin/time -v expam_limit -x 1.0 -t 10.0 -o classify.log expam classify -db [DATABASE NAME] --paired --alpha 0.1 -d [METAGENOME DIRECTORY] --out [OUTPUT_PATH] --taxonomy`

Supplementary Figures

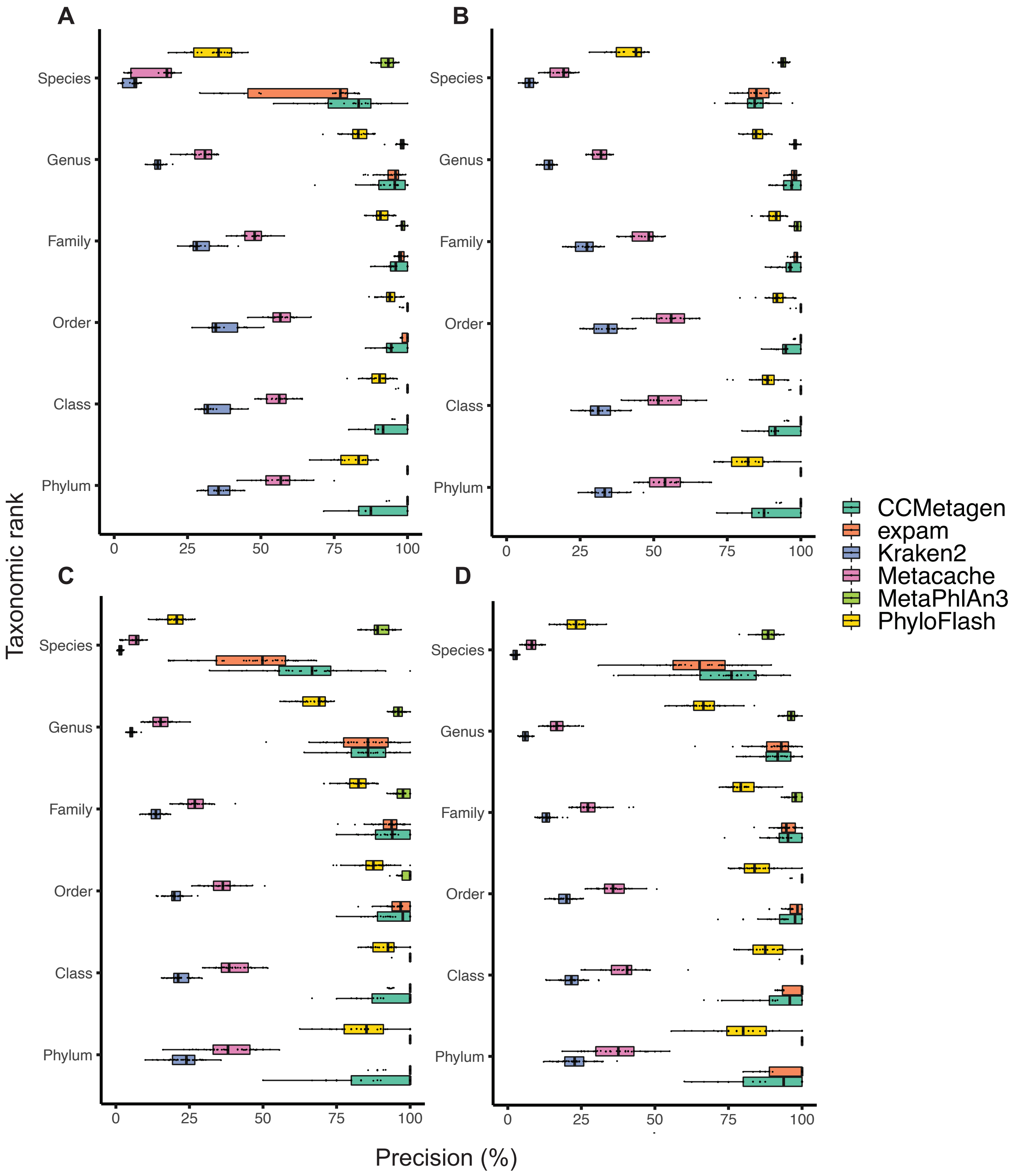

Figure S1: Taxonomic precision of metagenomic classifiers. Boxplots of precision distribution for classification of **A.** High complexity, false strain diversity (n= 30), **B.** High complexity, true strain diversity (n=30). **C.** Low complexity, false strain diversity (n=40), **D.** Low complexity, true strain diversity (n=40) *in silico* microbial communities.

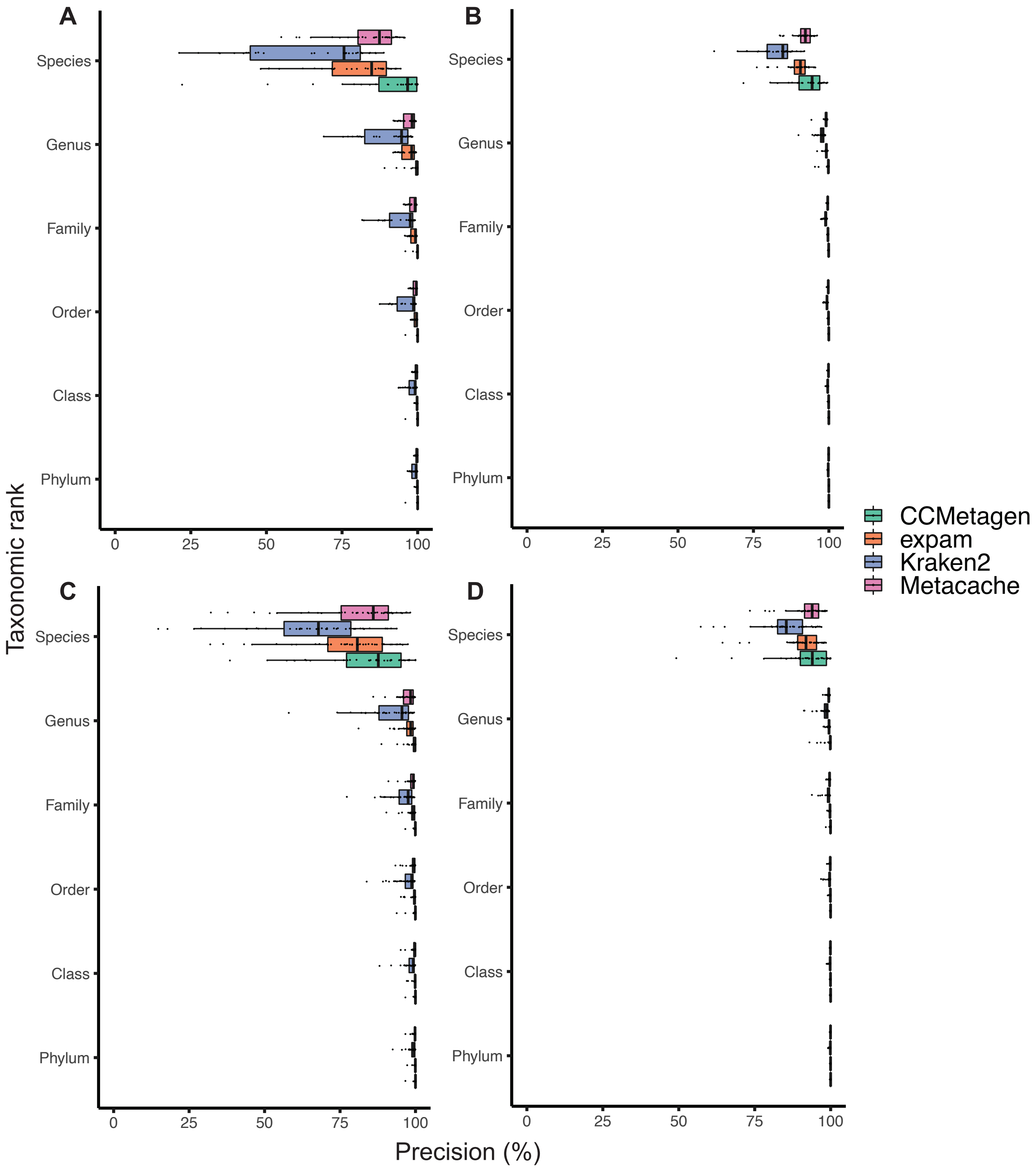

Figure S2: Read level precision of metagenomic classifiers. Boxplots of precision distribution for classification of four microbial community types. **A.** High complexity, false strain diversity (n= 30), **B.** High complexity, true strain diversity (n=30). **C.** Low complexity, false strain diversity (n=40), **D.** Low complexity, true strain diversity (n=40) *in silico* microbial communities.

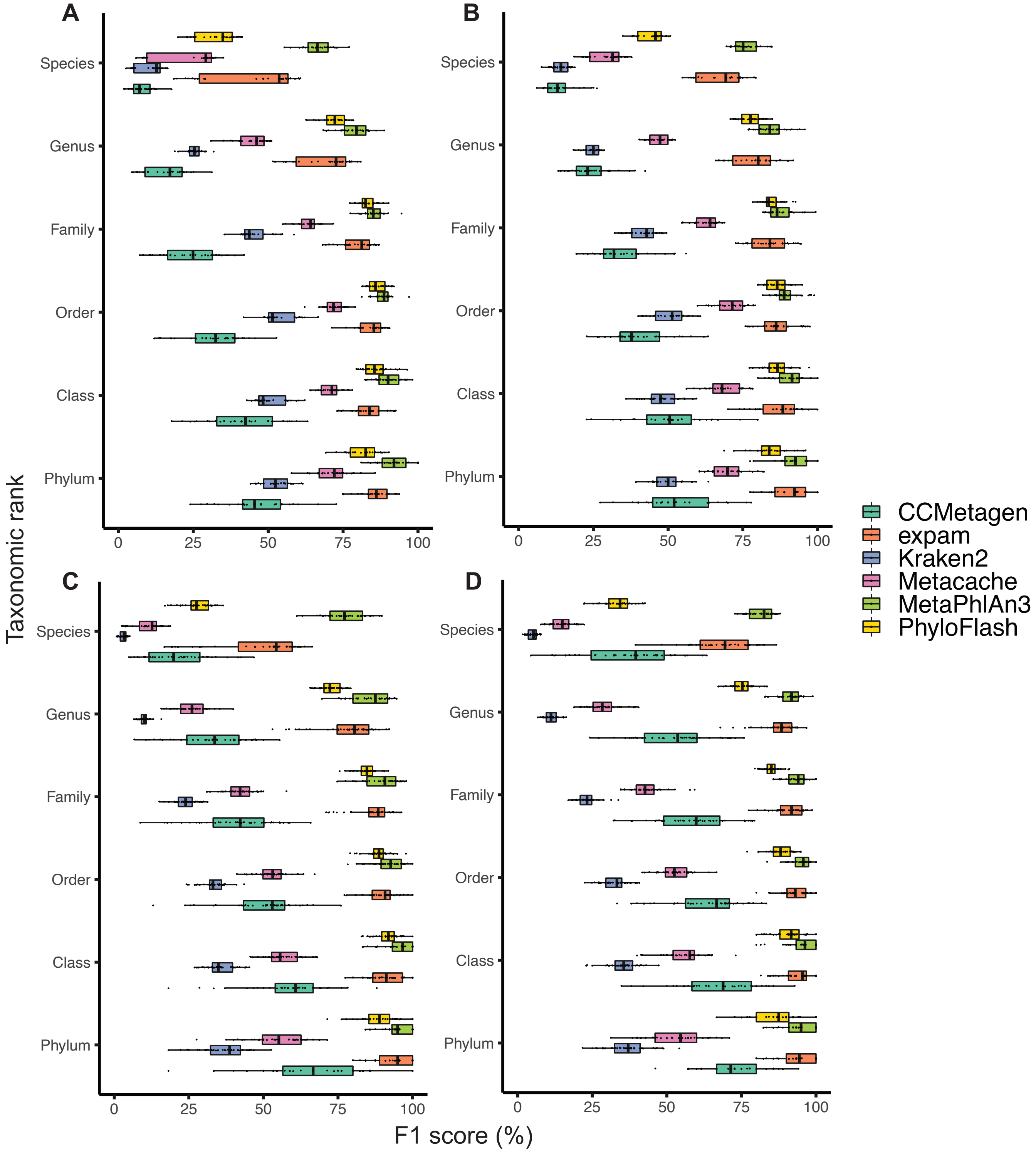

Figure S3: Taxonomic F1 score of metagenomic classifiers. Boxplots of F1 score distribution for classification of four microbial community types. **A.** High complexity, false strain diversity (n= 30), **B.** High complexity, true strain diversity (n=30). **C.** Low complexity, false strain diversity (n=40), **D.** Low complexity, true strain diversity (n=40) *in silico* microbial communities.

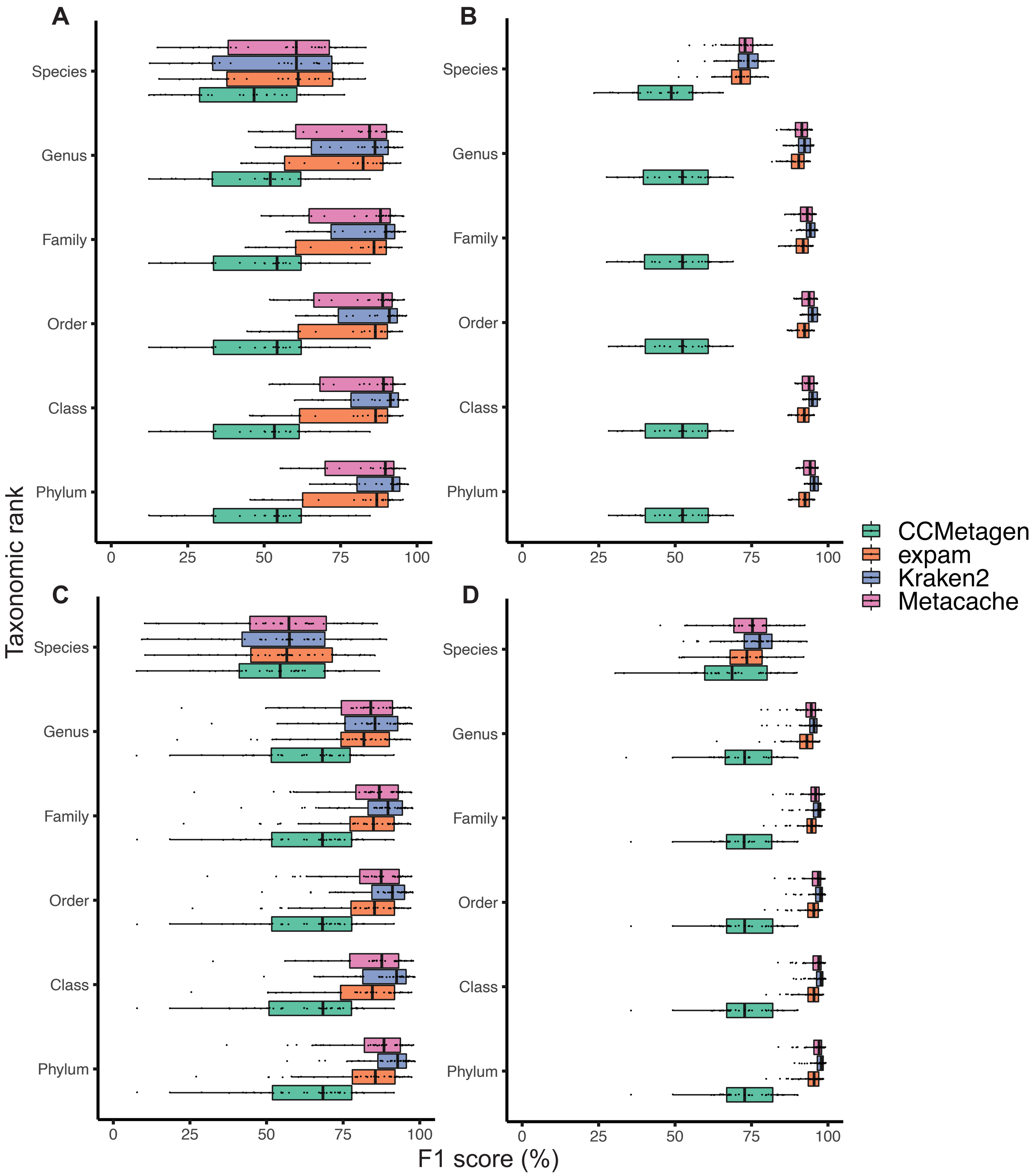

Figure S4: Read level F1 score of metagenomic classifiers. Boxplots of F1 score distribution for classification of four microbial community types. **A.** High complexity, false strain diversity (n= 30), **B.** High complexity, true strain diversity (n=30). **C.** Low complexity, false strain diversity (n=40), **D.** Low complexity, true strain diversity (n=40) *in silico* microbial communities.

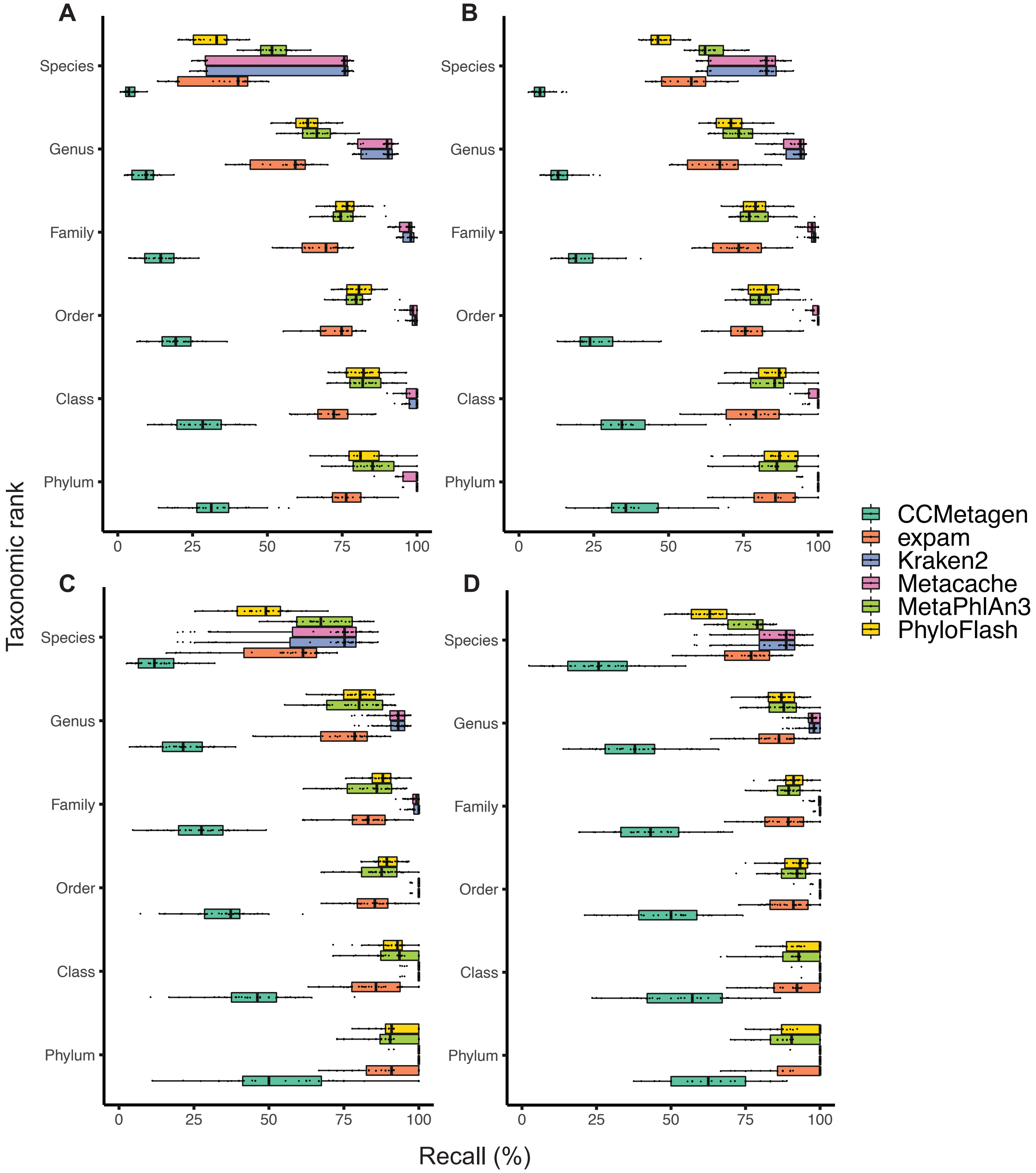

Figure S5: Taxonomic recall of metagenomic classifiers. Boxplots of recall distribution for classification of four microbial community types. **A.** High complexity, false strain diversity (n= 30), **B.** High complexity, true strain diversity (n=30). **C.** Low complexity, false strain diversity (n=40), **D.** Low complexity, true strain diversity (n=40) *in silico* microbial communities.

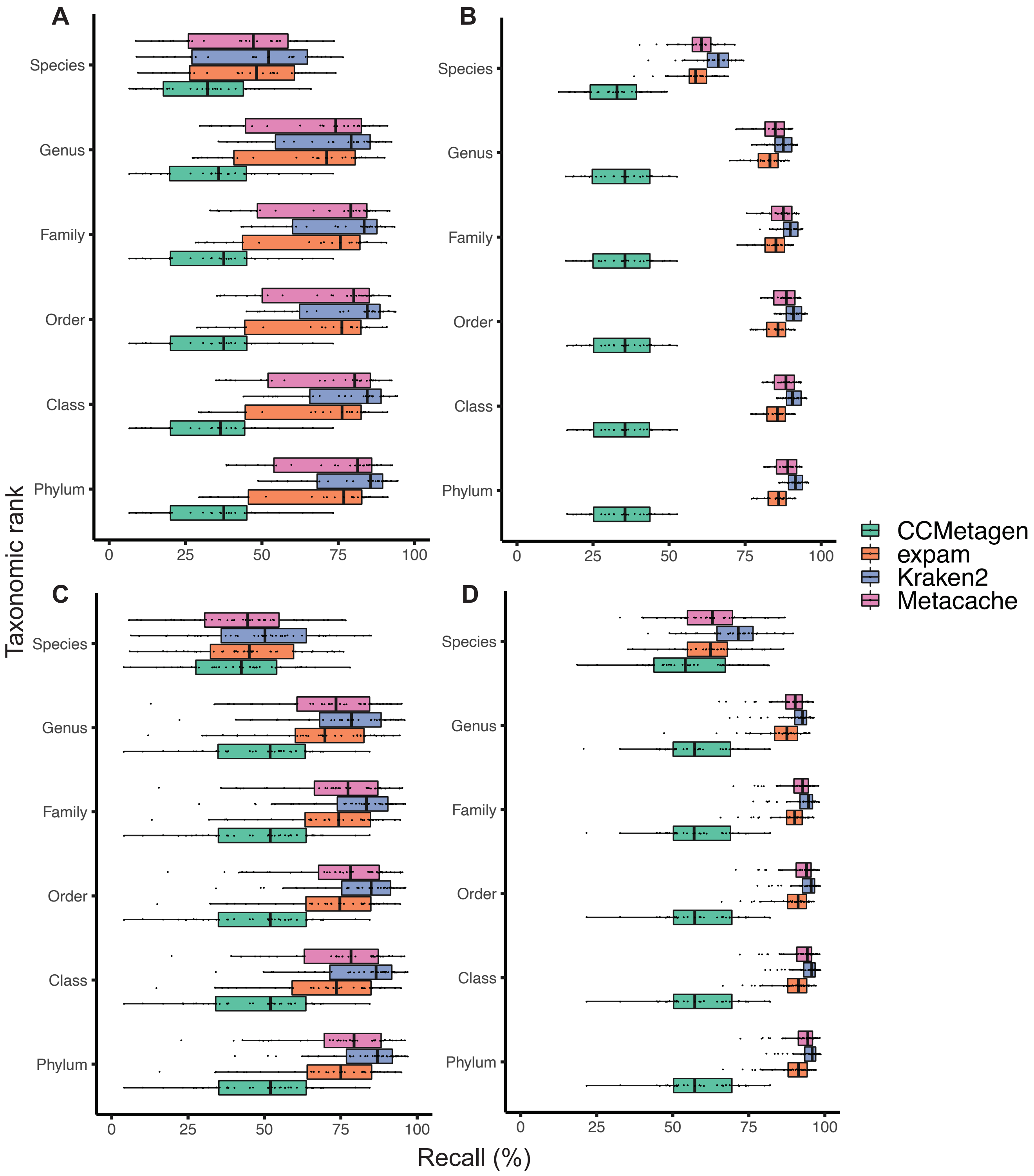

Figure S6: Read level recall of metagenomic classifiers. Boxplots of recall distribution for classification of four microbial community types. **A.** High complexity, false strain diversity (n= 30), **B.** High complexity, true strain diversity (n=30). **C.** Low complexity, false strain diversity (n=40), **D.** Low complexity, true strain diversity (n=40) *in silico* microbial communities.

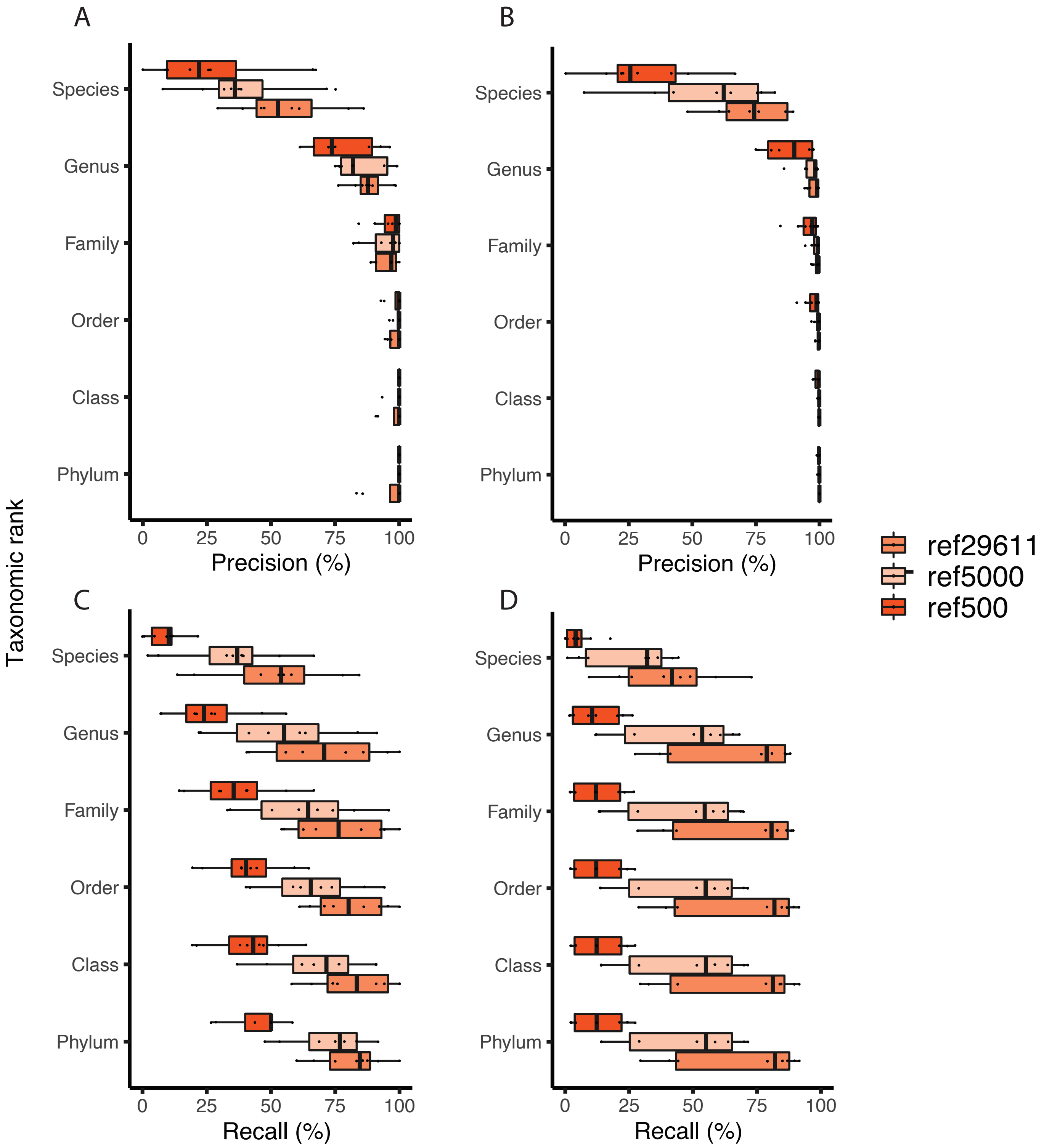

Figure S7: Read level and taxonomic precision and recall for varying number of reference genomes. Boxplots of precision and recall distribution for classification of eight *in silico* microbial communities, using three different databases: Ref500, Ref5000 and Ref29611. **A.** Taxonomic precision, **B.** Read level precision, **C.** Taxonomic recall, **D.** Read level recall.

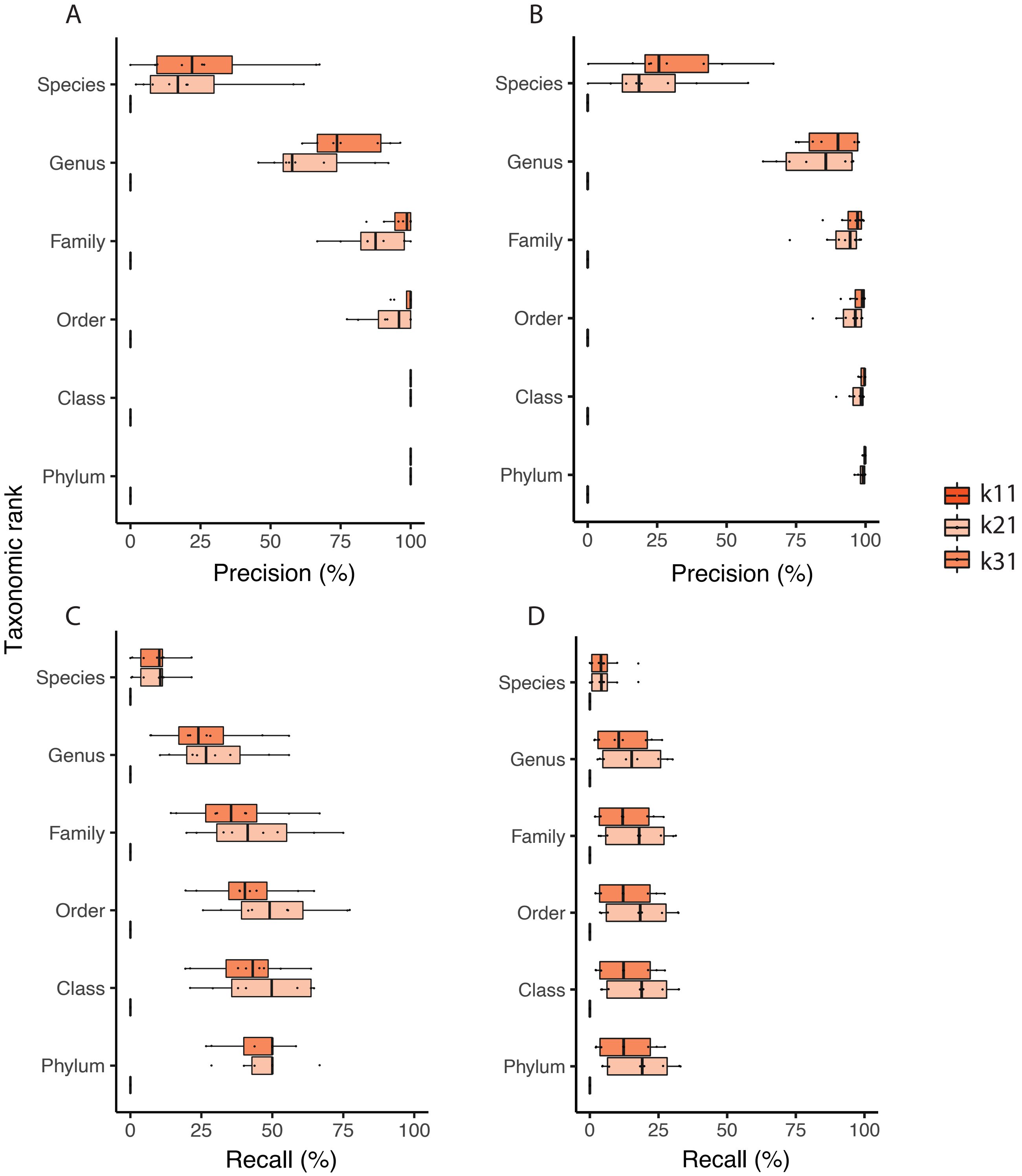

Figure S8: Read level and taxonomic precision and recall for varying database k-mer size. Boxplots of precision and recall distribution for classification of eight *in silico* microbial communities, using three different databases comprising Ref500 built with k=11, 21 and 31. **A.** Taxonomic precision, **B.** Read level precision, **C.** Taxonomic recall, **D.** Read level recall.

Supplementary Tables

Table S1: Metagenomic profilers used for comparison

Tool	Version
expam	1.0.4
CCMetagen	1.4.0
Kraken2	2.1.2
Metacache	2.0.0
MetaPhlAn	3.0.12
PhyloFlash	3.4

Table S2: ***In silico*** **mock communities used for benchmarking.**

Sample Name	Species Diversity	Strain Diversity	Number of Samples
High complexity, false strain diversity	500 ± 100	1 strain per species	30
High complexity, true strain diversity	500 ± 100	≤10 strains per species	30
Low complexity, false strain diversity	100 ± 25	1 strain per species	40
Low complexity, true strain diversity	100 ± 25	≤10 strains per species	40

Table S3: Total classification time and memory (mean ± standard deviation) for all 140 *in silico* mock communities.

Tool	User time (seconds)	Elapsed wall time (seconds)	Maximum memory used across all samples (kbytes)
Kraken2	84000 ± 1100	10570 ± 380	44230000 ± 330000
Metacache	391500 ± 7000	22400 ± 960	58497390 ± 660
MetaPhlAn3	550000 ± 1000	36258 ± 64	2814470 ± 170
CCMetagen	133510 ± 350	29150 ± 480	39219621 ± 20
PhyloFlash	177800 ± 1000	8782 ± 91	17628400 ± 8400
expam	2194000 ± 46000	101100 ± 1500	245936000 ± 31000

Table S4: Impact of number of reference genomes on database build (*k*=31, 32 processes)

Number of references	Build user time (seconds)	Build system time (seconds)	Maximum memory usage (kbytes)
500	6070	59	1440000
5000	86500	731	9920000
29611	1350000	26000	58940000

Table S5: Impact of *k* on database build (Ref500, 32 processes)

k Value	Database Size	Build user time (seconds)	Build system time (seconds)	Maximum memory usage (kbytes)
11	421 Kb	2590	24	827000
21	2.5 Gb	5550	56	1340000
31	4.6 Gb	6070	59	1440000

Table S6: Impact of number of reference genomes on classification runtime (*k*=31, 32 processes)

Number of References	Database Size	Classification user time (seconds)	Classification system time (seconds)	Maximum memory usage (kbytes)
500	4.6 Gb	55200	2800	9750000
5000	32 Gb	75700	3990	60800000
29611	123 Gb	83500	4020	245000000

Table S7: Impact of *k* on classification runtime (Ref500, 32 processes)

k Value	Classification user time (seconds)	Classification system time (seconds)	Maximum memory usage (kbytes)
11	24500	2880	4570000
21	64000	2970	9480000
31	55200	2800	9750000

Supplementary References

Huerta-Cepas, J. *et al.* (2016) ETE 3: Reconstruction, Analysis, and Visualization of Phylogenomic Data. *Mol. Biol. Evol.*, 33, 1635–1638.

Parks, D. *et al.* (2021) Evaluation of the Microba Community Profiler for Taxonomic Profiling of Metagenomic Datasets From the Human Gut Microbiome. *Front. Microbiol.*, 12.

Tisza, M. and Buck, C. (2021) A catalog of tens of thousands of viruses from human metagenomes reveals hidden associations with chronic diseases. *Proc. Natl. Acad. Sci. USA*, 118, e2023202118.
